# The Effect of Co_0.2_Mn_0.8_Fe_2_O_4_ Ferrite Nanoparticles on the C2 Canine Mastocytoma Cell Line and Adipose-Derived Mesenchymal Stromal Stem Cells (ASCs) Cultured Under a Static Magnetic Field: Possible Implications in the Treatment of Dog Mastocytoma

**DOI:** 10.1007/s12195-017-0480-0

**Published:** 2017-02-21

**Authors:** K. Marycz, M. Marędziak, D. Lewandowski, E. Zachanowicz, A. Zięcina, R. J. Wiglusz, R. Pązik

**Affiliations:** 1 Faculty of Biology, Wrocław University of Environmental and Life Sciences, Kożuchowska 5b, 50-631 Wrocław, Poland; 2 Faculty of Veterinary Medicine, Wroclaw University of Environmental and Life Sciences, Norwida 31, 50-375 Wrocław, Poland; 3 Institute of Materials Science and Applied Mechanics, Wroclaw University of Technology, Smoluchowskiego 25, 50-372 Wrocław, Poland; 4 Polymer Engineering and Technology Division, Wroclaw University of Technology, 50-370 Wrocław, Poland; 5 Institute of Low Temperature and Structure Research, PAN, Okólna 2, 50-422 Wrocław, Poland; 6 Centre for Advanced Materials and Smart Structures, Polish Academy of Sciences, Okolna 2, 50-950 Wrocław, Poland

**Keywords:** Mast cell tumors, Adipose derived mesenchymal stromal stem cell, Magnetic field, Oxidative stress, Nanoparticles, Magnetic properties

## Abstract

Cobalt manganese ferrite nanoparticles have application potential in the biomedical field, however there is limited information concerning the biological response. The aim of this work was to investigate the cytotoxic potential of cobalt-manganese ferrite nanoparticles in canine mastocytoma tumor cells (C2) and adipose-derived mesenchymal stromal stem cells (ASCs) cultured under a static magnetic field (MF). In this study, we investigated the viability and proliferation rate of ASC and C2 cells cultured with Co_0.2_Mn_0.8_Fe_2_O_4_ nanoparticles under 0.5T MF. We observed cells morphology and measured intracellular ROS generation. Thermal observations were used to characterize the thermotrophic cell behavior in different condition and RNA level of heat shock proteins and apoptotic genes was measured. Nanoparticles reduced cell viability, caused cell damage, i.e., through the formation of reactive oxygen species (ROS) and increased transcriptional level of apoptotic genes (Bcl-2, Bax, p53, p21). In addition, we have found that C2 mastocytoma cells cultured with metal oxide nanoparticles under MF exhibited unexpected biological responses, including thermotolerance and apoptotic response induced by the expression of heat shock proteins and ROS produced under a MF. Our results suggest that stimulation using MF and Co_0.2_Mn_0.8_Fe_2_O_4_ nanoparticles is involved in mechanisms associated with controlling cell proliferative potential signaling events. We can state that significant differences between normal and cancer cells in response to nanoparticles and MF are apparent. Our results show that nanoparticles and MF elevate the temperature *in vitro* in tumor cells, thereby increasing the expression of ROS as well as heat shock proteins.

**Electronic supplementary material:**

The online version of this article (doi:10.1007/s12195-017-0480-0) contains supplementary material, which is available to authorized users.

## Introduction

Mast cell tumors (MCTs) are the most common skin malignancy in dogs, comprising 16–21% of all cutaneous tumors diagnosed,^33^,^37^ mainly originating from neoplastic transformation of resident tissue mast cells or their progenitors.^34^

Canine MCTs demonstrate varying physiological behaviors, ranging from solitary benign masses, which can be cured with surgery alone, to a systemic and potentially fatal metastatic disease.^38^,^56^ In contrast to normal tissue, MCT microenvironment often lacks oxygen (hypoxia), is acidic (acidosis) and contains high levels of reactive oxygen species (ROS). Together, these factors promote tumor growth, spread and resistance to cancer therapies, leading to treatment failures. Strategies to normalize the tumor microenvironment have previously focused on targeting single factors or proteins associated with the abnormal microenvironment. Although dogs with a localized MCT are often cured by local therapy (surgery and/or radiation therapy), those with an inoperable primary mass or confirmed disseminated disease usually die.^4^,^56^

The use of both local and systemic therapies is limited by potential adverse effects, some of which can be reversible, cumulative or permanent in nature. Thus, there is a need to develop innovative therapies for MCTs with improved efficacy and higher therapeutic indices.

Magnetic hyperthermia in cancer therapy is one of the applications of metal oxide nanoparticles, where they are used in an external magnetic field as local heat dissipating agents.^17^ Metal oxide nanoparticles with a spinel structure have been extensively studied due to their potential magnetic storage applications and, more recently, as excellent candidates for contrast enhancement in magnetic resonance imaging (MRI),^15^,^59^ drug delivery,^9^,^32^ and other biomedical purposes.^28^,^50^ Several metal NPs that exhibited unique properties have been previously investigated.^1^,^63^,^64^ Research has focused in particular on cobalt, manganese and iron NPs in medical nanotechnology and biotechnology.^41^,^48^ Cobalt- and manganese-based NPs are attracting interest owing to their unique shape- and size-dependent properties and potential applications. Cobalt and manganese are one of the elements utilized in nanoparticle preparation for biomedical applications, as particles with appropriate physical properties are known to promote cellular uptake.^7^ The biocompatibility of nanoparticles, associated with attractive magnetic properties of certain ferrites, have led to the discovery of new applications of nanoparticles in the medical field,^23^,^45^,^54^ thus we explored the usefulness of Co_0.2_Mn_0.8_Fe_2_O_4_ ferrite nanoparticles for simultaneous modulation of MCT microenvironment.^44^

From the perspective of magnetic biology, it is reasonable to examine the cytotoxicity of magnetic nanoparticles (MNPs) in combination with MF exposure. MNPs are magnetized in the presence of MF and can potentially exert unidentified adverse effects.^2^ However, the use of a magnetic field has shown early promise in a number of *in vitro* and animal studies on adjunctive therapies or even a primary role in certain forms of cancer.^14^,^40^ An additional prospective advantage is that magnetic fields have the potential to cause less damage to normal tissue.^56^ In previous studies, we have tested the effect of a 0.5-T static magnetic field (MF) on normal cell growth. Our investigations concerning multiple parameters of mesenchymal stromal cells derived from adipose tissue (ASCs) indicated that the cells cultured under sMF increased their proliferation and secretory activity as well as the ability to differentiate into osteogenic lineage.^33^,^34^,^36^ However, quite a little studies relating to studying cellular toxicity of magnetic nanoparticles exposed to magnetic field has been conducted.^2^

The objective of this study was to investigate the influence of cobalt-manganese ferrite nanoparticles taken up by canine normal (adipose derived mesenchymal stem cells) and cancer (mastocytoma tumor) cells cultured under MF.

## Materials and Methods

### Nanoparticle Synthesis

The cobalt manganese ferrite nanoparticles were prepared accordingly to the previously described synthetic protocol.^42^ The Bradley microwave-stimulated method was used for the synthesis of Co_0.2_Mn_0.8_Fe_2_O_4_ nanoparticles. The preparation procedure in all cases was carried out in a quantity necessary to obtain 0.5 g of the product, using the following substrates: Co(acac)_2_ (99%, Alfa Aesar), Mn(acac)_2_ (99%, Alfa Aesar), and Fe(acac)_3_ (99%, Alfa Aesar), where acac is the acetylacetonate ligand. For instance, the preparation of Co_0.5_Mn_0.5_Fe_2_O_4_ nanoparticles involved the following quantities of reactants: 0.1131 g of Co(acac)_2_, 0.4455 g Mn(acac)_2_ and 1.5651 g Fe(acac)_3_. Organic metal complexes were transferred to 70 ml of acetophenone (99%, Sigma Aldrich, without further purification) forming an intense red solution and subsequently moved to a polytetrafluoroethylene (PTFE) container, which was placed in an Ertec^®^ Magnum V2 microwave reactor. The total reaction time was 90 min at 250 °C and 35 atm. A dark brown nanoparticle suspension was obtained as a product. The nanoparticles were washed with ethanol (96%, Avantor Performance Materials) using a laboratory centrifuge (10,000 rpm, 10 min) until the odor of acetophenone was no longer detectable, usually not less than six times. After removing the mother solution, nanoparticles were transferred to an ethanol/water mixture (90:10). The batch was divided in two, one part was dried in a laboratory drier for XRD and TEM characterisation, second portion of the colloid was used for characterization of nanoparticles effect on chosen cell lines.

Characterization of the Co_0.2_Mn_0.8_Fe_2_O_4_ crystal structure was done by X-ray powder diffraction (XRD) technique covering range of 2Θ angles between 5° and 100°, using a PANalytical X’Pert PRO X-ray diffractometer (Cu-*K*_α1_ = 1.54060 Å). Primary size and morphology of the nanoparticles were analyzed utilizing high-resolution transmission electron microscopy (HRTEM) with a Philips CM-20 Super Twin microscope, operated at 200 kV. Preparation of samples involved deposition of ethanol/water nanoparticle colloid droplet on the carbon covered copper microscope grid and drying under IR lamp. Mean particle size determination was performed using volume-weighted equation:

1 $$d_{\text{av}} = \sum {\frac{{n_{i} d_{i}^{4} }}{{n_{i} d_{i}^{3} }},}$$

where *d*_av_ stays for mean primary size of particles, *n*_*i*_ is the number of particles of given size and *d*_*i*_ is diameter of *i* particle. Hydrodynamic size was measured by using Nanosight NS 500 automated instrument using 405 nm line of laser diode as a light source backscattered further on measured objects. The sample for hydrodynamic size measurement was prepared by taking 1 ml of ethanol suspension containing nanoparticles and further on diluted with 19 ml of de-ionized water and transferred by peristaltic pumps to the sample chamber. Typically the starting concentration of nanoparticles in prepared suspension was around 500 μg/ml. Measurement was repeated at least three times and conducted with different dilution of particles to achieve satisfactory statistics and exclude errors connected with too high or too low amount of analyzed objects. From simultaneous measurement of the mean squared displacement of each particle tracked, the particle diffusion coefficient (*D*_t_) and hence sphere-equivalent, hydrodynamic radius (*r*_h_) can be determined using the Stokes–Einstein equation:

2 $$r_{\text{h}} = \frac{{K_{\text{B}} T}}{{6\pi \eta D_{\text{t}} }},$$

where *K*_B_ is Boltzmann’s constant, *T* is temperature and *η* is solvent viscosity (H_2_O). The analysis was done using Nanosight NTA 2.3 software.

Final concentrations of nanoparticles used in biological experiments was chosen based on dose response curve to see half maximal inhibitory concentration (IC50).

### Cells and Cell Culture

The canine mastocytoma C2 cell line^29^ was kindly donated by Dr. R. Elders (University of Edinburgh, UK) with permission from the originator (Prof. W. Gold, University of California, USA).

C2 mastocytoma were cultured in a medium consisting of Eagle’s minimal essential medium, supplemented with 5% FBS, 1% non-essential amino acids, 50 mg/ml gentamicin, 1% l-glutamine (all from Sigma Aldrich), whereas adipose-derived mesenchymal stem cells (ASCs) were cultured in Dulbecco’s Modified Eagle’s Medium (DMEM) with 4500 mg/l glucose, supplemented with 10% FBS and 1% penicillin/streptomycin solution.

Mesenchymal stem cells were isolated from subcutaneous adipose tissue (2 g) collected from the dogs’ tail bases, according to the standard surgical procedure and ethical standards. All experimental procedures were approved by the 2nd Local Ethics Committee of Wroclaw University of Environmental and Life Sciences (Chelmonskiego 38C, 51-630 Wroclaw, Poland; decision No. 84/2012). Procedures were performed under local anesthesia using 2% lignocaine (Polfa S.A., Poland). After harvesting, specimens were placed in a sterile Hank’s balanced salt solution (HBSS) containing 1% antibiotic–antimycotic solution (Sigma-Aldrich, Poland) to remove contaminating debris and red blood cells. Tissue fragments were finely minced and digested with collagenase at a concentration of 1 mg per ml of culture medium (Sigma-Aldrich, Poland). In order to facilitate enzyme action, the samples were placed into a cell culture incubator (temperature 37 °C, 5% CO_2_) for 30 min. Next, samples were centrifuged at 1200×*g* for 10 min. After centrifugation, the supernatants were discarded, whereas the pellets of the stromal-vascular adipose fraction (SVF) containing ASC precursors were resuspended in culture medium.

Prior to the experiment, the purity of ASCs was confirmed by means of flow cytometry (absence of the CD34 hematopoietic marker, and the CD45 lymphocyte common antigen, as well as the presence of mesenchymal markers: CD90, CD105). In addition, the cell’s ability to differentiate into chondroblasts, osteoblasts, and adipoblasts was assessed.

All cell handling procedures were performed in a sterile laminar flow hood. All cell culture incubation steps were performed at 37 °C and 5% CO_2_.

### Cell Proliferation Assay

For the analysis of cell proliferation, C2 and ASC cells were inoculated into 24-well plates at an initial concentration 2 × 10^4^ per well. The cells were inoculated in a 0.5 mL volume of culture medium per well. After 4-h incubation, the MNP suspension medium was added in the final concentration of 1 µg/ml. A well without an MNP sample was used as a control. Both ASC and C2 cells were incubated in MF and control conditions. After 24, 48 and 72 h of incubation, the effect of different NP concentrations was determined. Cell proliferation factor (PF) was evaluated using the resazurin-based assay (TOX-8, Sigma Aldrich) according to the manufacturer’s instructions. Briefly: culture media were replaced with a medium containing 10% of resazurin-based dye and incubated for 2 h. Afterwards, the supernatants were collected and subjected to absorbance measurement using a spectrophotometer (SPECTRO StarNano, BMG Labtech) at 600 nm wavelength, with a 690 nm distraction of background absorbance. To evaluate the proliferation rate and the number of viable cells, a standard curve was calculated with the absorbance directly proportional to the number of cells.

### Evaluation of Cell Morphology

The analysis of cell morphology and growth pattern was performed on the 7th day using an inverted fluorescence microscope (Axio Observer A1, Zeiss) and a scanning electron microscope (SEM; EVO LS15, Zeiss). The evaluation of ASC morphology included the analysis of nuclei and cytoskeleton development. The nuclei were stained using diamidino-2-phenylindole (DAPI) and cytoskeleton was stained using atto-565-labeled phalloidin. Fluorescent staining was performed after fixation with 4% paraformaldehyde. Procedures involving fluorescence staining were performed in accordance with the manufacturers’ instructions and methods described previously.^25^,^27^,^35^

Photographs were acquired using a Power Shot Camera (Cannon).

### Thermal Analysis

Thermal images were acquired using a FLIR B335 thermographic digital camera. The camera was powered on for couple of minutes before the measurements. The camera was placed on a horizontal surface at a 0.5-m distance perpendicular to plate surface. All images were acquired in the same room at a stable ambient temperature (21 °C).

### Determination of Intracellular ROS Generation

To evaluate oxidative stress levels, the cells were cultured in a normal growth media without phenol red for 2 days. The production of reactive oxygen species (ROS) was estimated fluorometrically using an H2DCF-DA reagent (Life Technologies). Before exposure, the cells were washed with phosphate-buffered saline and incubated with 5 μM H2DCF-DA for 30 min at 37°C. The fluorescence of H2DCF-DA was detected at excitation and emission wavelengths of 485 and 530 nm, respectively.

### Apoptosis Assessment

After 7 days of culture, the cells were fixed and terminal deoxynucleotidyl transferase dUTP nick-end labeling (TUNEL) assay was performed following the manufacturer’s instructions (In Situ BrdU- Red TUNEL Assay Kit, Abcam). The number of viable and dead cells was evaluated with Cellstain Double Staining Kit (Sigma Aldrich). Viable cells were stained with Calcein-AM and emitted green fluorescence, whereas the nuclei of dead cells were stained orange with propidium iodide. The cells were observed using an epifluorescence microscopy and all procedures were conducted in accordance with the manufacturer’s instructions. Apoptosis was quantified by counting the number of TUNEL-positive cells in five random microscopic fields (×40); the percentage of dead cells was also calculated.

### qRT-PCR Analysis

Total RNA was extracted using the TRIreagent (Sigma Aldrich**)** and the phenol–chloroform method.^10^ Purity and quantity of RNA was assessed using nanospectrophotometry (VPS Biowave II). Genomic DNA digestion and cDNA synthesis were performed using PrimeScript kit (Takara, Clontech). The qRT-PCR mixture contained 150 ng of cDNA, 500 nM of forward and reverse primers, and SensiFast SYBR & Fluorescein Kit SYBR Green PCR Master Mix (Bioline). The PCR reaction profile consisted of initial enzyme activation at 95 °C for 10 s, followed by 40 cycles of denaturation at 95 °C for 15 s, annealing for 30 s with temperature dependent on primer sequences and extended at 72 °C for 30 s with a single fluorescence measurement. The series of cycles were followed by a melt curve analysis to ensure reaction specificity. The expression level of each gene was normalized to the housekeeping gene—GAPDH. Subsequently, the relative gene expression (Qn) was calculated in relation to the GAPDH gene. Primer sequences used in individual reactions are listed in Table 1.

**Table 1:** Primers sequences used in experiment.

Gen	Primer sequence 5′–3′		Product length (bp)	NCBI reference sequence
GAPDH	F:	GATTGTCAGCAATGCCTCCT	**198**	XM_003435649.3
	R:	GTGGAAGCAGGGATGATGTT		
Bax	F:	ACCAAGAAGCTGAGCGAGTGTC	365	NM_001003011.1
	R:	ACAAAGATGGTCACGGTCTGCC		
Bcl-2	F:	TTCTTTGAGTTCGGTGGGGT	164	NM_001002949.1
	R:	GGGCCGTACAGTTCCACAA		
p21	F:	GAGACGGTGGCTTGGAGAG	**185**	XM_532125.5
	R:	CACCTGCAGCTCCTCCG		
p53	F:	GTACCGGTGACTGCAATGGA	494	NM_001003210.1
	R:	ACAACCTCGGTCACGAACTC		
HIFα	F:	CTCAAATGCAAGAACCTGCTC	86	NM_001287163.1
	R:	TTCCATACCATCTTTTGTCACTG		
HSP70	F:	AGCACCTTTCCTTTCGCAGA	536	NM_001003067.2
	R:	CCTCGGCGATCTCCTTCATC		

### Statistical Analysis

All experiments were performed with triplicate or more. Statistical analysis was performed using GraphPad Prism 5 software (La Jolla, USA). Differences between groups was determined using One way Anova with Bonferroni’s Multiple Comparison Test. Differences with a probability of *p* < 0.05 were considered significant.

## Results

### Structure and Morphology of Co_0.2_Mn_0.8_Fe_2_O_4_ Nanoparticles

**Figure 1:**
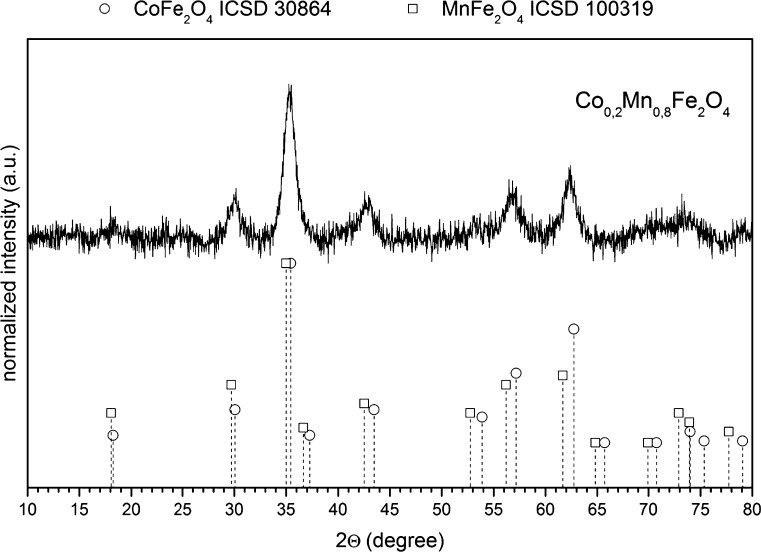
XRD pattern of the Co_0.2_Mn_0.8_Fe_2_O_4_.

Crystal structure of the prepared Co_0.2_Mn_0.8_Fe_2_O_4_ was measured using XRD technique (Fig. 1). As it can be seen the recorded XRD pattern consists only of characteristic and broad peaks matching perfectly with the reference standards of the ferrite spinel family (CoFe_2_O_4_ ICSD 30864, MnFe_2_O_4_ ICSD 100319).

**Figure 2:**
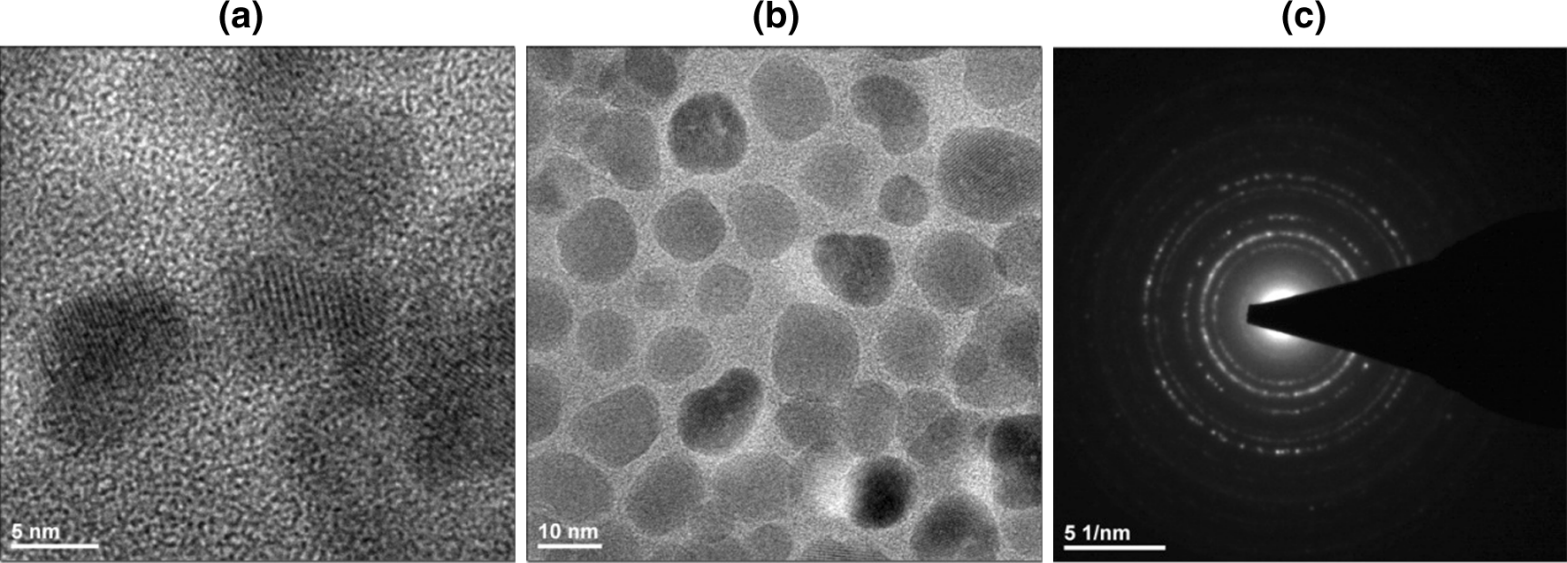
Representative TEM (**a**, **b**); SAED (**c**) images of the Co_0.2_Mn_0.8_Fe_2_O_4_ nanoparticles.

In order to estimate the primary particle size of the Co_0.2_Ni_0.8_Fe_2_O_4_ nanoparticles TEM images were taken (Figs. 2a, 2b). The mean particle size was around 10 nm with narrow size distribution ±2 nm. Particles were not agglomerate and adopted regular shapes. SAED images showed the appearance of well-developed and spotty rings with positions and distances close to the reference standards (Fig. 2c). In the context of biological applications determination of the particle size by TEM technique is not enough since so-called primary particle size in most cases has nothing to do with the state of the particles suspended in water. Therefore it is mandatory to provide more convincing data regarding hydrodynamic size. The state of the particle in water suspensions is determined by two main mechanisms contributing to the formation of agglomerates of particles. The former one involves Van der Waals interactions describes attractive inter-particle forces and the latter one through binding of adsorbed molecules^43^ Thus DLS technique was utilized to estimate hydrodynamic size which in this particulate case was around 58 nm (see Fig. 1 in supplementary).

### Immunophenotyping and Multipotency Assay

**Figure 3:**
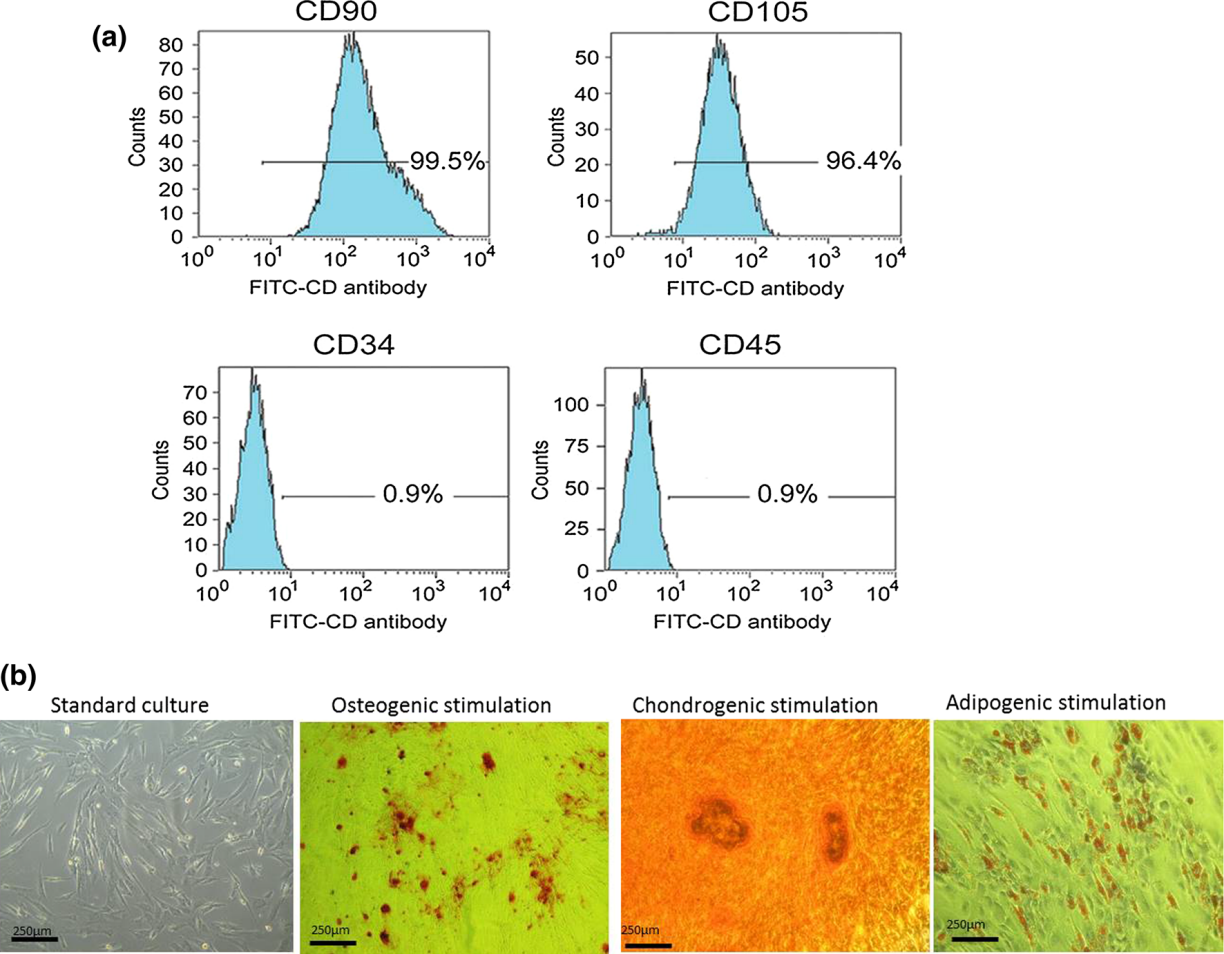
(**a**) Immunophenotyping of Canine ASCs using FITC dye-conjugated antibodies to identify different cell-surface differentiation markers. (**b**) Mineralized nodules formed by differentiated cells after incubation in osteogenic medium (stained with Alizarin red S), cartilage extracellular matrix formed by differentiated cells after incubation in chondrogenic medium (stained with Safranin O) and intracellular lipid vacuoles found in differentiated cells after incubation in adipogenic medium (stained with Oil Red O).

The isolated cells presented typical for ASC features, including (i) plastic-adherent growth, (ii) expression of CD90 and CD105 and (iii) the lack of expression of CD45 and CD34 surface antigens (Fig. 3a). The obtained ASCs differentiated into osteogenic, chondrogenic and adipogenic lineages, which was verified by specific stainings (Fig. 3b).

### Evaluation of Cell Viability and Morphology

**Figure 4:**
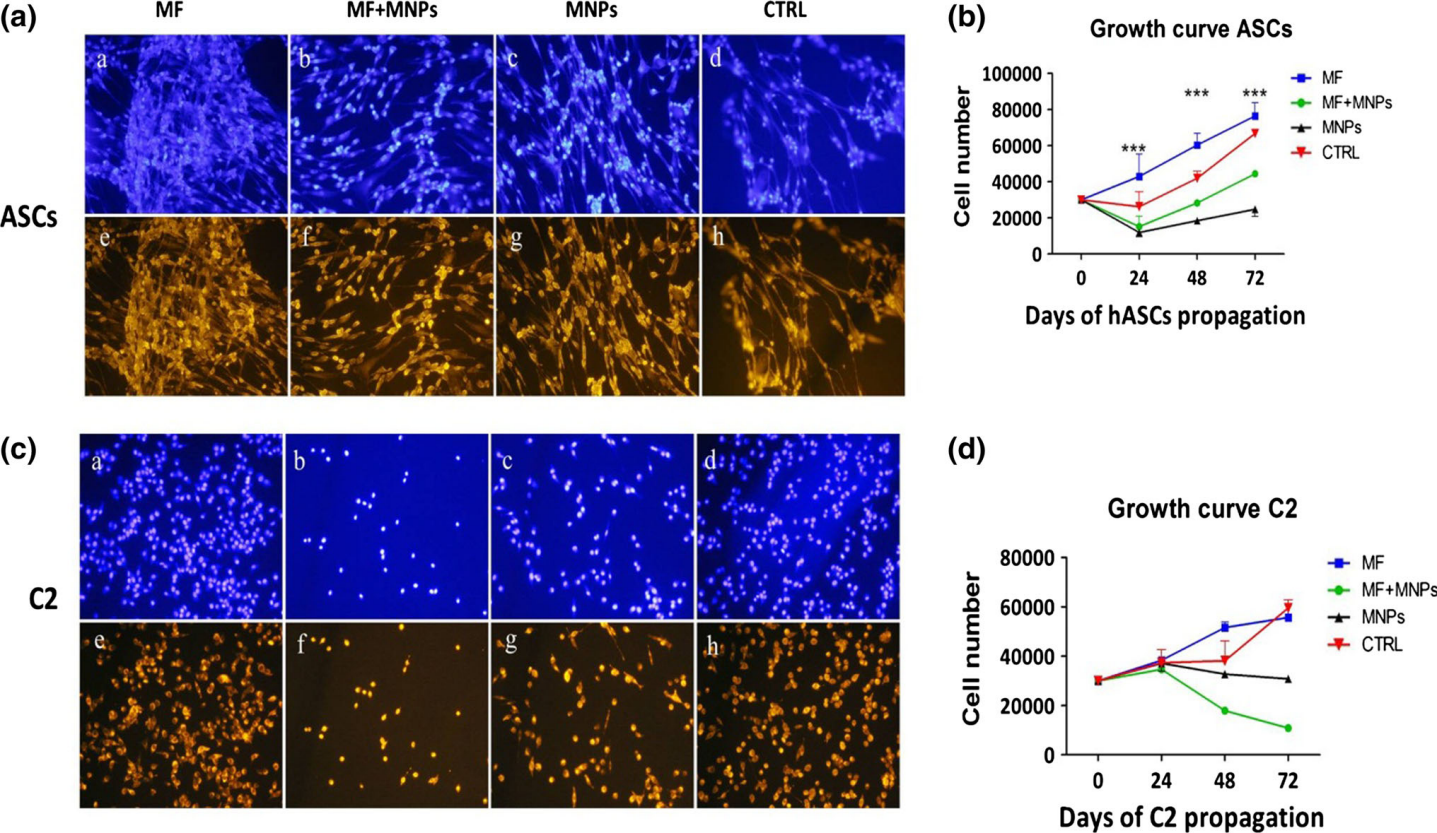
(**a**) Canine ASCs **(c)** Canine C2 mastocytoma: nuclei (**a**–**d**) and actin filaments (**e**–**h**) stained with DAPI (blue) and phalloidin (red), respectively. (**b**) ASCs and (**d**) C2 proliferation rate measured at different time points. Results expressed as mean ± SD. *p < 0.05.

The proliferation factor (PF) of canine adipose derived mesenchymal stem cells and mastocytoma C2 cells was evaluated during 72 h of cell culture (Figs. 4b and 4d). The proliferation assay have shown that the magnetic field significantly affected the proliferation rate of the cells, as those cultured under MF reached the highest proliferation rate. Stimulation of ASCs with MNPs resulted in an inhibition of proliferation at all time points examined. Interestingly, the cells exposed to MF with MNP treatment (MF + MNPs) were characterized by a lower PF than the control group. The viability of cells was assessed after seven days of culture.

The resazurin based measurements indicated a reduction in the *proliferation rate* of canine C2 mastocytoma cells co-incubated with MNPs and in the presence of both- MNPs and MF compared to the control and MF conditions applied separately (Fig. 4d). As expected, the highest proliferation rate was observed in cells cultured under MF and control conditions.

We observed a proper fibroblast-like morphology of ASCs in all investigated cultures (Fig. 4a). The cells demonstrated uniform growth patterns. ASCs treated with MF formed tightly packed monolayer characteristic of mesenchymal stem cells, whereas ASCs cultured with MNPs adopted a multipolar shape. C2 mastocytoma cells had rounded nuclei or spindle morphology. The cells cultured with MNPs predominantly exhibited an adherent, spindle-shaped morphotype (Fig. 4c).

### Thermal Analysis

**Figure 5:**
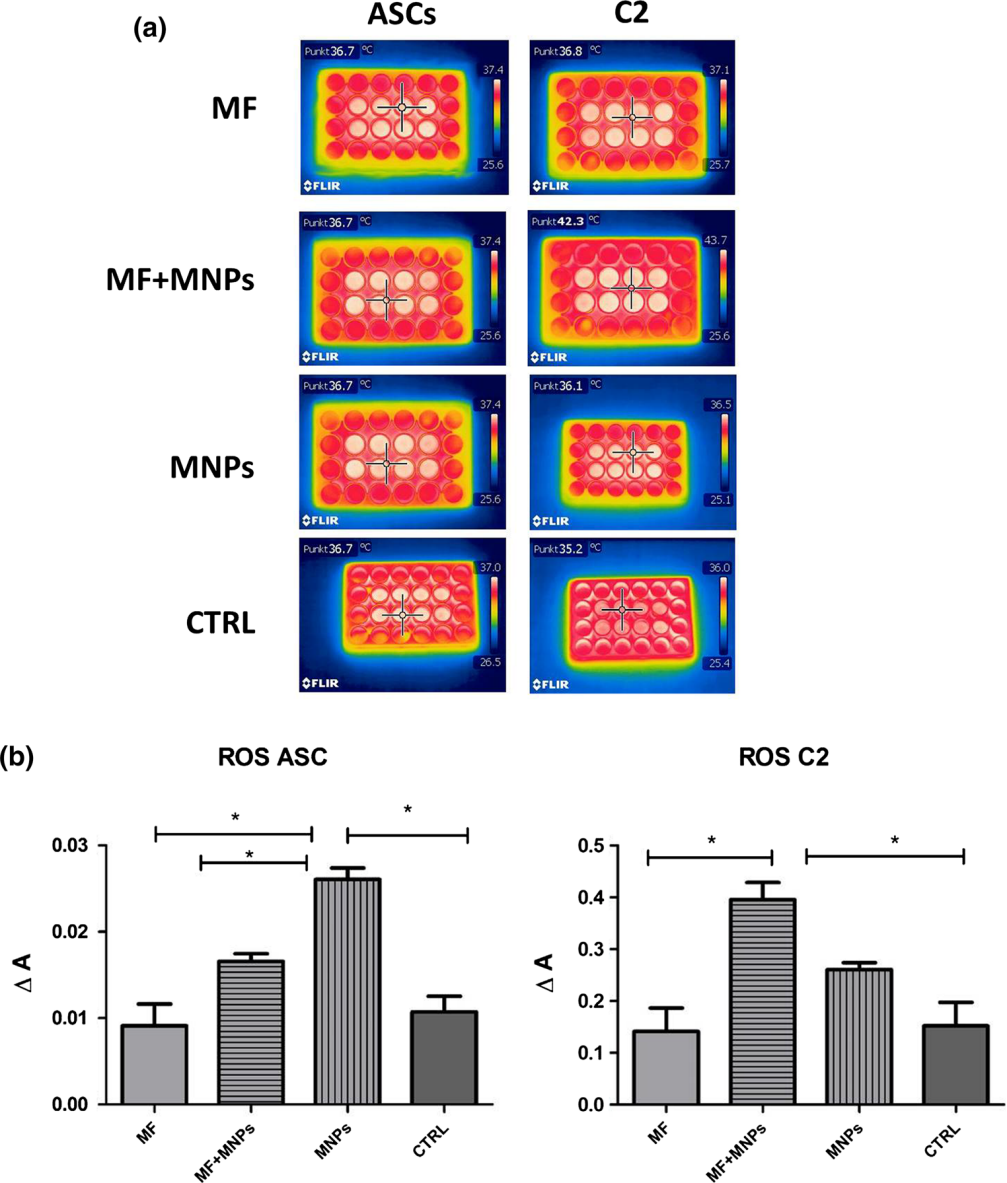
(**a**) Representative thermal imaging pictures of ASCs and C2 cells treated with MNPs and MF or control cells cultured in 24-well plates. Temperature profiles indicated on images. (**b**) Reactive oxygen species in cell cultures. Results expressed as mean ± SD. *p < 0.05.

Thermal observations were used to characterize the thermotrophic cell behavior in different conditions (Fig. 5a). Thermographic camera for detecting and quantifying temperature-induced transitions showed a clearly higher temperature of C2 cells cultured with MNPs under MF (MF + MNPs), and the measured temperature was 42.3 °C. The cells cultured under control conditions did not show an increased temperature (36.8 °C). Interestingly, all ASC cultures had similar temperatures, at about 37 °C, even those co-cultured with MF and MNPs.

### Oxidative Stress Factor Analysis

To determine whether MNPs and MF treatment could affect the level of oxidative stress factors, we evaluated the level of ROS after 72 h of culture (Fig. 5b). When comparing ROS concentrations between ASCs and C2, the upregulation in tumor cells was apparent. Nevertheless, comparisons within the groups considered (ASCs and C2) indicated that the lowest concentration of ROS was detected in cells exposed to MF. In the case of ASCs, significant differences were observed in cells treated only with MNPs, whereas in C2, a significantly higher ROS concentration was detected in cells co-cultured with MF and MNPs.

### Magnetic Field and Nanoparticle-Induced Apoptosis in ASC and C2 Cultures

**Figure 6:**
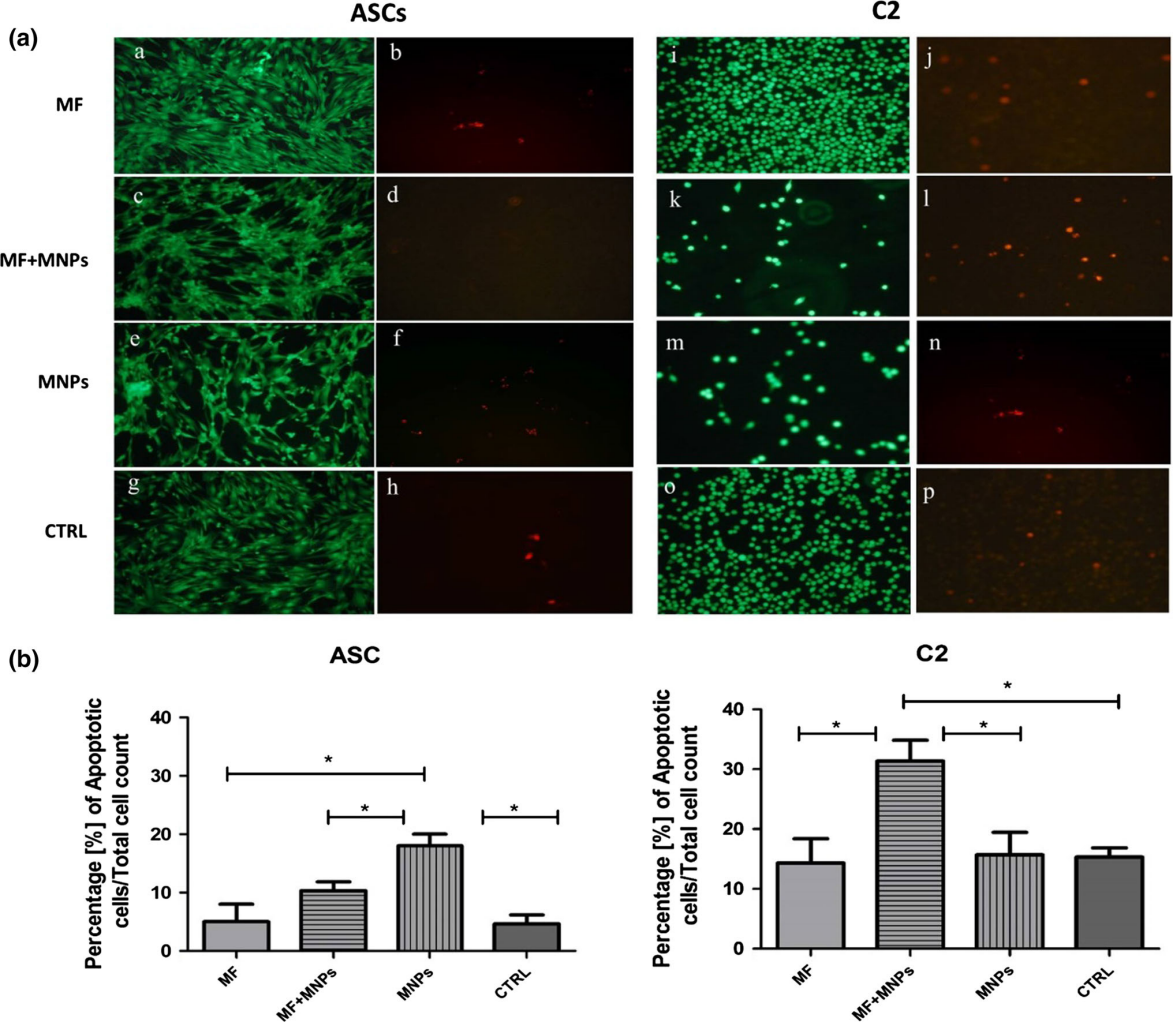
**(a)** Images showing the results of calcein (green) and propidium iodide (red) staining of ASCs and C2. **(b)** TUNEL assay – percentage of apoptotic cells.

Magnetic properties of the Co_0.2_Mn_0.8_F_2_O_4_ nanoparticles were studied in detail by us showing purely superparamagnetic properties.^42^ The exposure of C2 cells to both MF and MNPs resulted in a significant apoptotic cell death, as demonstrated by the calcein/propidium iodide staining (Fig. 6a) and quantification of apoptotic nuclei in the TUNEL assay (Fig. 6b). The number of PI-positive C2 cells was increased in cells cultured with both MNPs and MF in comparison to control group. The percentage of PI-positive ASCs was the highest in cells cultured with the addition of MNPs. The cells cultured with MNPs and MF exhibited a similar level of PI-positive cells as those cultured with MF only. The results of the TUNEL assay strongly correlated with the viable-dead staining. The highest percentage of apoptotic ASCs was observed in the MNP-treated groups, whereas in case of C2 cells, in the group treated both with MF and MNPs.

**Figure 7:**
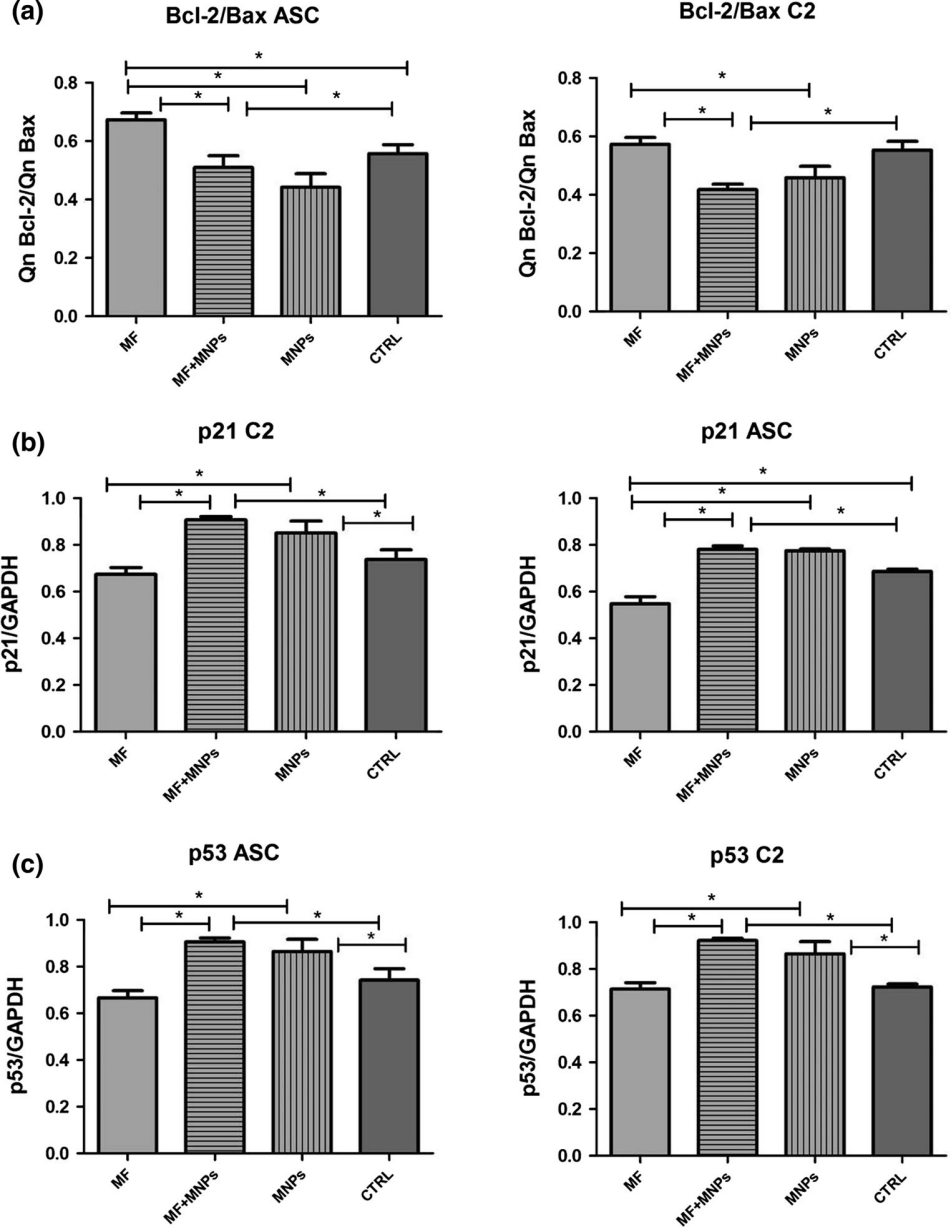
(**a**) Quantitative analysis of Bcl-2; (**b**) p21; (**c**) p53 at the mRNA levels.

Quantitative analysis of Bcl-2/Bax gene expression (involved in mitochondrial apoptotic pathway) at the mRNA level (Fig. 7a) revealed that only ASCs under MF increased Bcl-2/Bax ratio when compared to control, whereas C2 under MF were not different in comparison to control. Other test groups had comparable levels of Bcl-2/Bax ratio. The level of p21 (Fig. 7b) and p53 (Fig. 7) transcripts was increased in cultures treated with MF + MNPs in ASC and C2 cultures.

**Figure 8:**
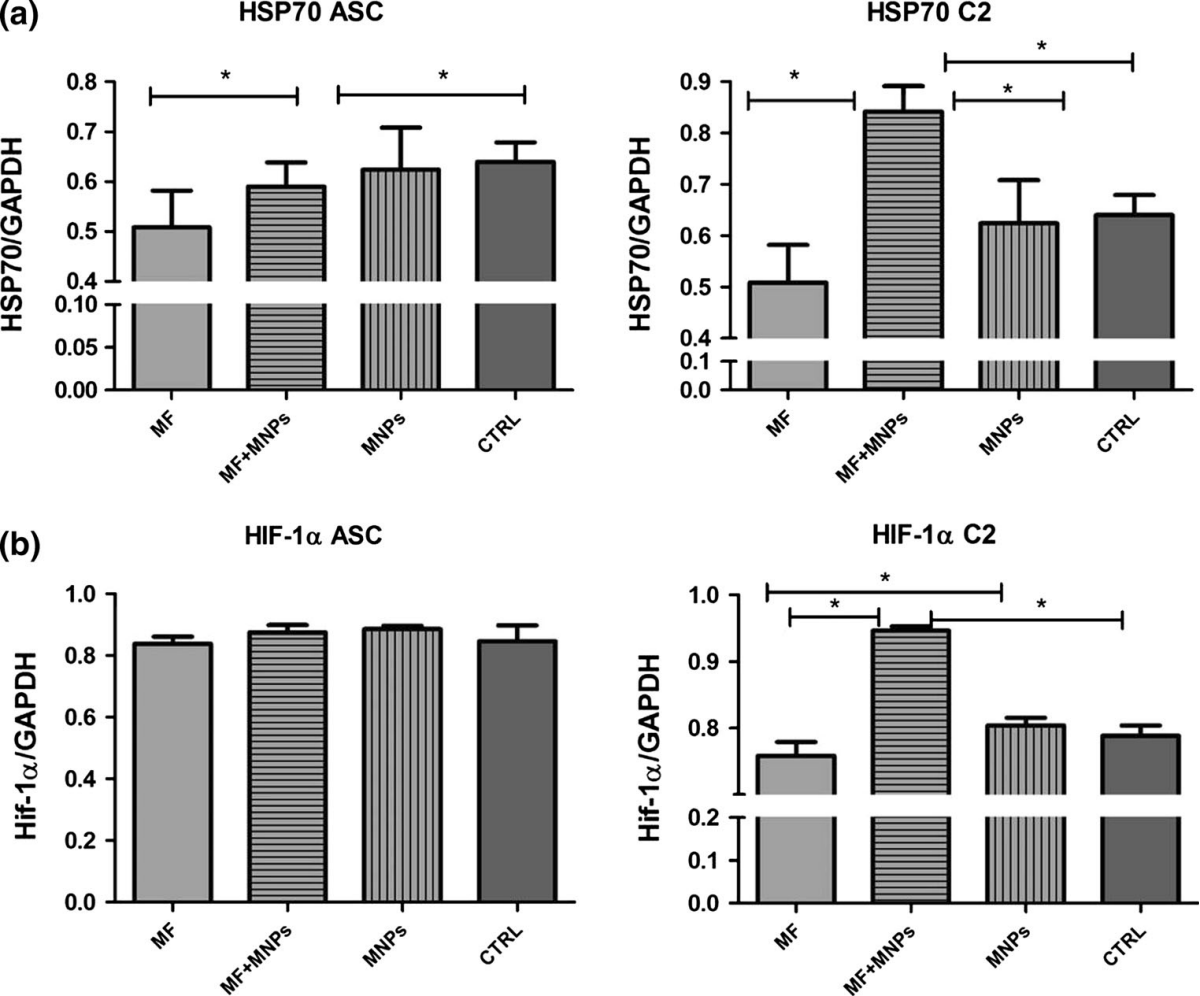
(**a**) Quantitative analysis of HSP70; (**b**) Hif-1α at mRNA levels.

We have compared the expression of heat shock protein 70 (Hsp70) (Fig. 8a) and hypoxia inducible factor-1 alpha (Hif-1α). The expression of Hif-1α was comparable in all ASC experimental groups, whereas HSP70 was the highest in cells cultured with MNPs and in control group. We observed significantly different results in C2 cells. The expression of HSP70 and was two-fold higher in cells cultured with MF and MNPs. Interestingly, the cells cultured with only MF and/or MNPs had a similar, lower expression of both genes compared to control.

## Discussion

Dogs are at risk of cutaneous MCT, which accounts for up to 21% of all skin tumors.^62^ The treatment of MCT by surgery or radiation is straight forward in the majority of cases, but creating an accurate cure is more challenging.^22^ Magnetic nanoparticles are a promising approach to cancer therapy. Therefore, in our study we have proposed the use of cobalt-manganese ferrite nanoparticles for this purpose. The results of several studies have shown that nanoparticle therapies are based on the principle that the magnetic particles can generate heat through hysteresis losses under a magnetic field. Gordon *et al*.^19^ first proposed the concept of intracellular hyperthermia using dextran magnetite (Fe_3_O_4_) nanoparticles. They administered magnetite nanoparticles to Sprague–Dawley rats bearing mammary carcinomas, and showed that MF-induced heating occurred in their *in vivo* experiments. Jordan *et al*.^24^ conducted several comprehensive *in vitro* studies on magnetic fluid hyperthermia using dextran magnetite nanoparticles. Our results suggest that cobalt-manganese ferrite nanoparticles are potentially an effective tool for hyperthermic treatment of dog skin mastocytoma. This approach, in addition to killing tumor cells with heat, does not induce normal cell (ASCs) response.^46^ The function of proper cells cells in the damaged tissues may also be modified by the transfer of cell receptors, cytoplasmic proteins, and mRNAs from surrounding cells by microvesicles (MVs), which are spherical structures in which a part of the cell cytoplasm enriched for mRNA, miRNA, and functional proteins is encapsulated by cell membrane. We have performed a detailed analysis of the factors that were upregulated and released from tumor cells. In our study we observed different cytotoxic reactions of ASCs and C2 cells on MF and MNP stimulation. Different plasma proteins on the cell surface may cause different patterns of phagocytic uptake, and thus different levels of cytotoxicity.^49^ Moreover, on the basis of electrostatic interactions with the ASC cell membrane, MNPs can be expected to have a high adhering capacity, whereas MNPs stimulated only C2 cells without adhering to their surface. Mast cells are known to either secrete or rapidly release mediators into the local microenvironment after activation.^39^ A significantly elevated temperature in C2 observed in our study could be the effect of MNP movement in response to different MF and cell activation. Thus, tumor mast cells were stimulated to degranulation, similarly as by stimuli induced during physical injury or pathogen-related agents,^39^,^53^ causing local inflammation with a higher temperature.

We also found that MNPs and MF exerted their cytotoxic effect via the induction of apoptosis in the C2 mastocytoma. These data are consistent with previous reports that used HL-60 cells.^18^ We further demonstrated that the induction of apoptotic cell death through an elevated temperature by combining MNPs and MF in culture was modulated by the antioxidant capacity of the ROS degrading enzyme system in the cells. In line with these experiments, the induction of apoptosis was significantly different between C2 and ASC cells. The partial resistance of ASCs was correlated with significantly lower ROS levels than in C2 cells. ROS production by mitochondria during apoptosis has already been considered by several investigators.^26^,^30^,^31^ Oxidative stress is believed to be one of the causative factors of apoptosis in pathogenesis and aggressiveness of most cancers.^12^ A moderate increase in ROS level often induces cell proliferation, whereas excessive amounts of ROS induce apoptosis.^13^ Our results demonstrated that MNPs under MF induced apoptosis in C2 cells through increased production of ROS. Previous findings showed that increased temperature induced ROS generation and apoptosis in various cell types,^20^,^52^ but cellular sources of ROS remain controversial. The findings of Wang *et al*. were consistent with the established role of mitochondria as key sources of ROS generation through the electron transport chain.^61^ Several lines of evidence have indicated that heat stress can induce ROS production in different cell types.^20^,^25^,^51^ The precise mechanisms of how the temperature increases mitochondrial ROS levels remain undetermined. The reasons may be manifold. On one hand, higher temperature may increase mitochondrial ROS generation. During normal mitochondrial function, a small percentage of electrons from the electron transport chain reduces oxygen to superoxide. During mitochondrial dysfunction, this leak of electrons is increased.^61^ Tissier *et al*. have previously reported that mild hypothermia preserves mitochondrial function and reduces mitochondrial ROS generation.^57^ In contrast, dysfunction in mitochondrial respiration may increase the formation of ROS in mitochondria.^16^ Swerdlow *et al*. also reported that the enhanced production of mitochondrial ROS was linked to mitochondrial dysfunction.^55^ On the other hand, the temperature may result in a decreased antioxidant capacity in mitochondria.

To gain insight into the mechanism of apoptosis, different proteins with regulatory functions in apoptotic pathways were investigated. Bcl-2, p53, p21 proteins and heat shock proteins (HSPs) are important general regulators of apoptotic cell death.^6^,^8^,^11^ Proliferation factor measurements indicated decreased cells division which suggested that MF and MNP could impact on cells and induce cell senescence or cellular apoptosis. To confirm hypothesis we measured p21 and p53 gene expressions which monitor DNA replication and cell division. Whereas it is known that Bcl-2 proteins affect the apoptotic pathway via interactions with the mitochondrial membrane potential, HSPs seem to exert their apoptotic effect at the mitochondrial level as well.^3^,^60^ In our experiment, Bcl-2 expression was at a similar level in C2 cells compared with ASCs (both stimulated by MF and MNPs). Thus, it seems that these members of the Bcl-2 family are not involved in the induction of apoptosis by MF. In contrast, hyperthermia-dependent induction of HSPs directly correlated with the apoptotic effect in ASC and C2 cells. However, mRNA level of HSPs was significantly increased after exposure to MF + MNPs in C2 cells; the induction of ASCs was markedly diminished. This differential HSP expression in C2 and ASC cells implies that the hyperthermia-induced increase of HSPs is dependent on ROS accumulation. Taken together and consistently with earlier reports,^21^,^58^ our data support the notion that the induction of HSP is dependent on the cellular redox status. Moreover, ROS have been implicated as an oxygen sensor, and thereby linked to the regulation of HIF-1 under limited oxygen supply. Moreover, growing evidence have demonstrated that HIF-1 also responds to non-hypoxic stimuli, such as hormones, growth factors, peptides and cytokines,^5^ and ROS were also suggested to play a critical role in HIF-1 expression under non-hypoxic conditions.^47^

In conclusion, we can state that significant differences between normal and cancer cells in response to MNPs and MF are apparent. Our results show that MNPs and MF elevate the temperature *in vitro* in tumor cells, thereby increasing the expression of ROS as well as HSPs and Hif-1α.

## Electronic supplementary material

Below is the link to the electronic supplementary material.

Supplementary material 1 (JPEG 646 kb)
