# Supplementary figures and images for: The Effect of Co0.2Mn0.8Fe2O4 Ferrite Nanoparticles on the C2 Canine Mastocytoma Cell Line and Adipose-Derived Mesenchymal Stromal Stem Cells (ASCs) Cultured Under a Static Magnetic Field: Possible Implications in the Treatment of Dog Mastocytoma
Source: Cell Mol Bioeng. 2017 Feb 21;10(3):209–22. doi: 10.1007/s12195-017-0480-0 (PMC5434168; doi:10.1007/s12195-017-0480-0)

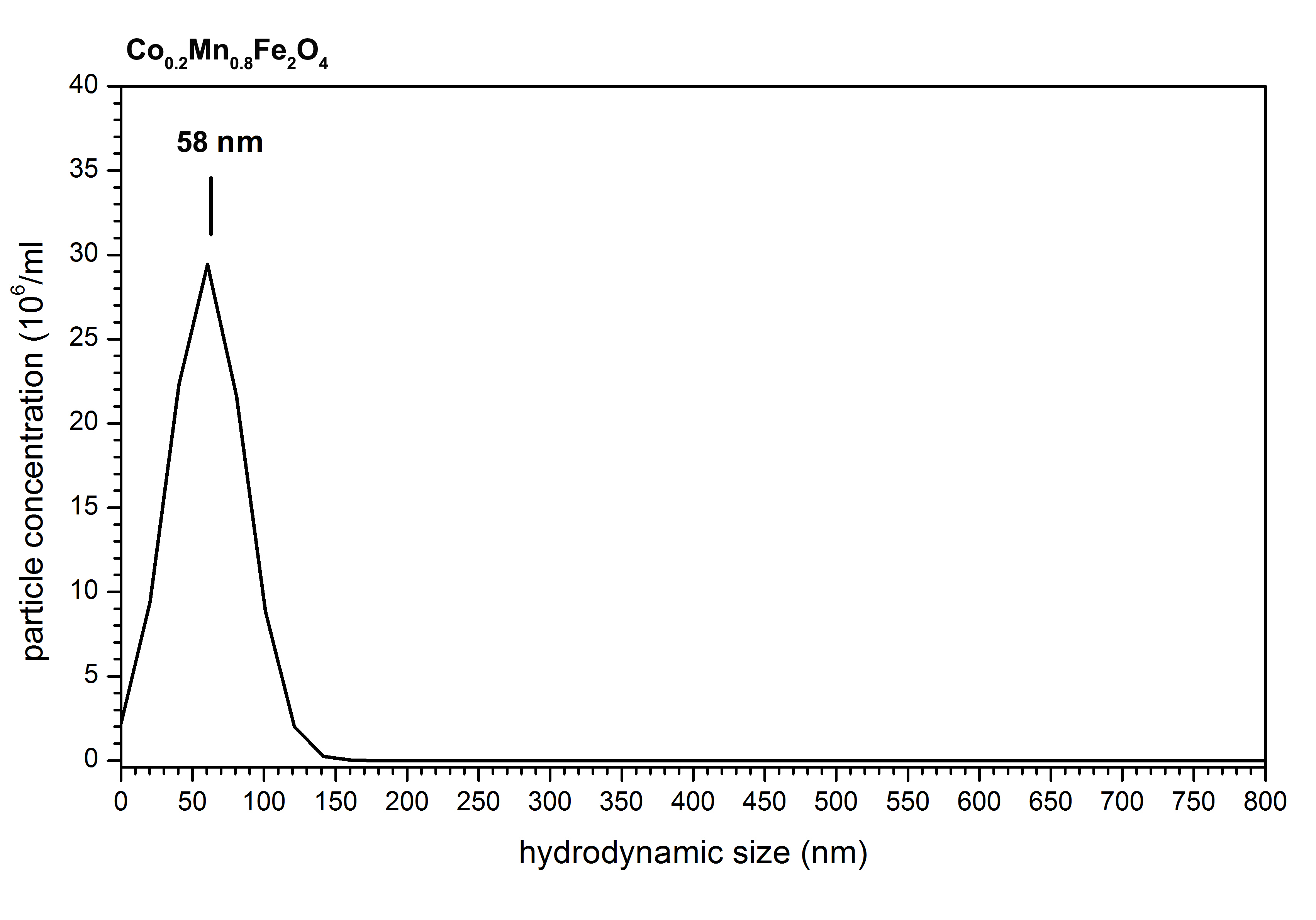

Supplement: Supplementary file 1 — Supplementary material 1 (JPEG 646 kb) [file 12195_2017_480_MOESM1_ESM.jpg]
